# Assessing changes in the availability and readiness of health facilities to provide modern family planning services in Bangladesh: Insights from Bangladesh Health Facility Surveys, 2014 and 2017

**DOI:** 10.1371/journal.pone.0334520

**Published:** 2025-11-14

**Authors:** Rajon Banik, Syed Toukir Ahmed Noor, Abu Bakkar Siddique, Md. Sabbir Ahmed Mayen, Anindita Saha, Ashiquzzaman, Lubna Hossain, Abu Sayeed, Nondo Saha, Md. Akib Al- Zubayer, Md. Lutful Kader, Ema Akter, Md. Abu Bakkar Siddique, Anisuddin Ahmed, Sahar Raza

**Affiliations:** 1 Maternal and Child Health Division, International Centre for Diarrhoeal Disease Research, Bangladesh (icddr, b), Dhaka, Bangladesh; 2 Department of Public Health and Informatics, Jahangirnagar University, Savar, Dhaka, Bangladesh; 3 Department of Statistics, Shahjalal University of Science and Technology, Sylhet, Bangladesh; 4 Global Health and Migration Unit, Department of Women’s and Children’s Health, Uppsala University, Uppsala, Sweden; Khulna University, BANGLADESH

## Abstract

**Background:**

Modern family planning plays a vital role in reducing unintended pregnancies, a major reproductive health issue worldwide. Access to modern family planning services is essential for empowering women to have greater control over their reproductive health and rights. In Bangladesh, there remains an unmet need for modern family planning services among reproductive-aged women. Assessing the capacity of health facilities to address these unmet needs for modern family planning is crucial.

**Objectives:**

The objective of this study was to assess the changes in the availability and readiness of health facilities to provide modern family planning services in Bangladesh between 2014 and 2017, and identify factors associated with facility readiness.

**Methods:**

We performed a secondary analysis of cross-sectional data from Bangladesh Health Facility Surveys (BHFS) conducted in 2014 and 2017. Availability was determined based on whether a facility offered at least one modern family planning method, and facility readiness was measured following the Service Availability and Readiness Assessment (SARA) manual. Descriptive statistics with 95% confidence intervals (CIs) were reported, and Poisson regression models were used to identify factors associated with health facility readiness.

**Findings:**

The percentage of facilities offering modern family planning services increased significantly from approximately 81% (95% CI: 78, 85) in 2014 to 89% (95% CI: 87, 91) in 2017. The availability of oral pills, injectables, and male condoms increased over this period, while the availability of long-acting reversible contraceptives (LARCs) slightly decreased, and permanent methods (PMs) remained nearly unchanged. The overall mean readiness score of health facilities declined slightly, from about 54 (95% CI: 52, 56) in 2014 to 51 (95% CI: 50, 53) in 2017. Upazila Health Complexes and Maternal and Child Welfare Centers had significantly higher readiness compared to District Hospitals in 2017. Facilities that performed routine quality assurance activities, ensured 24-hour staff coverage, maintained a system for reviewing clients’ feedback, and provided family planning services regularly demonstrated significantly higher readiness to provide modern family planning services in both 2014 and 2017. Regional disparities were also observed; facilities in rural areas had significantly lower readiness than those in urban areas, and facilities from the Rangpur division showed significantly higher readiness compared to those in Dhaka in both survey years.

**Conclusion:**

The findings indicate a significant increase in the availability of health facilities offering modern family planning services in Bangladesh; however, a slight decline has been observed in their overall mean readiness score. Ensuring an adequate provision of equipment and supplies, expanding access to LARCs and PMs, and improving staff capacity through regular training are essential. Furthermore, strengthening quality assurance activities and investing in rural facilities are required for improving the facility readiness and advancing progress toward achieving SDG 3.7 targets of universal access to modern family planning services in Bangladesh.

## Introduction

Approximately 121 million unintended pregnancies take place annually, which accounts for 73 million abortions every year [1]. Low- and middle-income countries (LMICs) account for 92% of unintended pregnancies, leading to approximately 55 million unplanned births [1]. According to the World Health Organization (WHO), 6 out of every 10 unplanned pregnancies end up being induced abortions, making it a frequent consequence of unintended pregnancy [2]. Complications related to induced abortion, particularly unsafe abortions, contribute to more than 16 thousand maternal deaths and an estimated disability-adjusted life years (DALYs) of 1.1 million every year [3]. Unintended pregnancies also impact newborn development indicators and heighten the likelihood of low birth weight [4]. Additionally, it contributes to poorer maternal bonding and mental health challenges in children [5], which can persist into adulthood [6,7]. Unintended pregnancy is also responsible for maternal psychological disorders such as postpartum depression, anxiety, stress, and lower satisfaction with life [4,8,9].

Family planning is defined as the use of contraceptive methods or other birth control methods that allow individuals and couples to anticipate and attain their desired number of children and the spacing and timing of their births [6]. Modern family planning refers to the use of modern contraceptive methods such as female sterilization, male sterilization, intrauterine contraceptive devices, implants, injectables, pill, male condoms, female condoms, emergency contraception, and the lactational amenorrhea method, whereas traditional methods include rhythm (calendar), withdrawal, and folk methods [7]. In 2022, the use of modern family planning methods prevented about 150,000 maternal deaths, 29 million unsafe abortions, and over 141 million unintended pregnancies [8]. Family planning is a prerequisite for women’s autonomy and gender equality, a fundamental human right, and a key strategy of alleviating poverty [9]. Access to modern family planning services plays a crucial role in averting unintended pregnancies and sexually transmitted infections, accelerating economic growth, and population control [10,11]. Moreover, modern family planning is one of the most affordable public health interventions, offering significant long-term returns. Every US$1 invested in addressing the unmet need for modern family planning methods can generate up to US$120 in annual benefits [12].

Approximately 1.1 billion women of reproductive age required family planning services in 2021 worldwide, and among them, 874 million women used modern contraceptive methods, while 92 million relied on traditional methods [13]. Since 1990, the number of modern contraceptive users worldwide has nearly doubled, highlighting the higher effectiveness of modern family planning methods over traditional methods [13]. However, the unmet need for modern family planning rose from 147 million in 1990–164 million in 2021 [6]. In Bangladesh, substantial progress has been made in modern family planning services over the past decade, with the use of modern methods of contraceptives rising from 52% in 2011 to 55% in 2022, though 9% of women still use traditional methods [14]. A persistent high total fertility rate (2.3 births per woman) [14], a stagnant contraceptive prevalence rate of 64% among currently married women aged 15–49 [15], and a 10% unmet need for modern family planning methods [14] make it challenging for Bangladesh to achieve SDG target 3.7 by 2030, which aims for universal access to sexual and reproductive health services, including family planning [16]. Evidence indicates that to expand the coverage of modern family planning services, health facilities must ensure the availability and readiness to provide a regular supply of a wide range of modern contraceptive methods [17,18]. Over the years, the Ministry of Health and Family Welfare (MOHFW) has implemented several facility-level initiatives, including improving contraceptive availability, enhancing service delivery, and training healthcare providers. Although monitoring and evaluation are essential for success, this area remains underdeveloped and requires significant improvement [19].

There is currently limited evidence regarding the capacity of health systems in Bangladesh to provide modern family planning services. Evidence from some LMICs, such as Nepal [20], Pakistan [21], and Uganda [22] indicate that health facilities often have low readiness to provide family planning services, which can negatively affect service provision and quality. In Bangladesh, existing research has provided valuable insights into the coverage, socio-demographic factors, and disparities in family planning service uptake [22–24]. For example, one study investigated regional differences in health facility readiness for long-acting reversible contraceptives (LARCs) and permanent methods (PMs), and another study assessed the impact of facility-level factors on LARCs usage [18,25]. However, no study has comprehensively assessed the health system’s capacity to provide modern family planning methods, including short-acting reversible contraceptives (SARCs), LARCs, and PMs. Therefore, this study aimed to examine the changes in the availability and readiness to provide modern family planning services using data from the Bangladesh Health Facility Surveys (BHFS) 2014 and 2017. Additionally, it sought to identify factors associated with the readiness of health facilities to provide modern family planning services.

## Materials and methods

### Data source

This study used data from two rounds of nationally representative BHFS conducted in 2014 and 2017. The BHFS gathers information on the availability of services and readiness of health facilities for family planning, maternal and child health (MCH), infectious and non-communicable diseases, staff availability, basic services, logistical support (such as essential medications, equipment, and laboratory services), and the infection control practices of government, non-profit, NGO, and private health facilities. The National Institute of Population Research and Training (NIPORT) conducted the BHFS 2014 and BHFS 2017 with technical support from ICF, USA [7,26]. To monitor and ensure the quality of fieldwork, NIPORT received assistance from the International Centre for Diarrheal Disease and Research, Bangladesh (icddr,b). The surveys were financially supported by the United States Agency for International Development (USAID) and the Government of Bangladesh. Standardized questionnaires from the Service Provision Assessment (SPA) component of USAID’s Demographic and Health Survey (DHS) Program were used to collect data on service availability and facility readiness.

### Data collection

BHFS 2014 and BHFS 2017 used two types of questionnaires: the facility inventory questionnaire and the questionnaire for healthcare provider interviews [7,26]. For this study, we included all variables from the facility inventory questionnaire (*Family planning section*) and one variable on staff training from the health care provider questionnaire. The BHFS 2014 data was gathered from May to July, while the BHFS 2017 data was gathered from July to October. The BHFS methodology, sampling, data collection instruments, and methods are available in detail elsewhere [7,26].

### Sample and sampling technique

**Fig 1:**
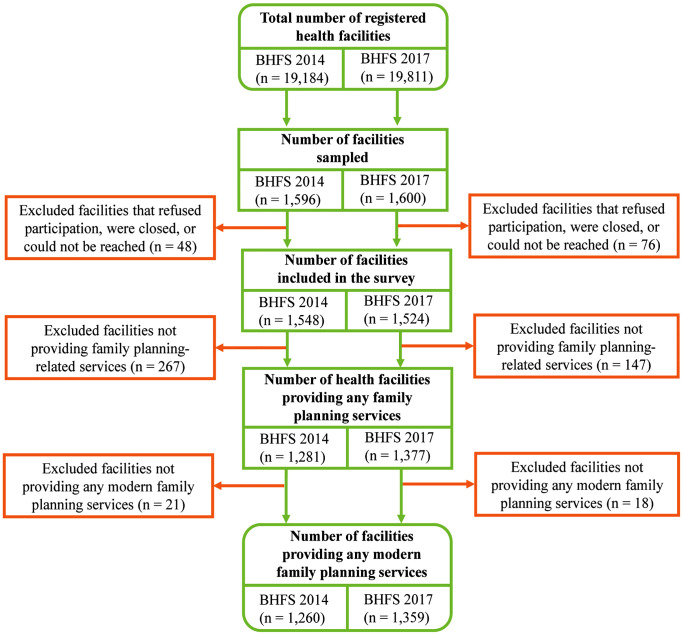
Flowchart of the sample selection procedure from BHFS 2014 and 2017.

The sampling process and data collection techniques employed in the BHFS are available on the DHS website [27,28]. Both the BHFS 2014 and BHFS 2017 utilized a stratified random sampling design to ensure representative data on health facilities across Bangladesh. The sampling frames for the surveys consisted of 19,184 registered health facilities in 2014 and 19,811 in 2017, compiled by the NIPORT and the MOHFW. These comprehensive frames ensured representation from a wide range of health facilities, including six different kinds of public facilities, private hospitals with 20 beds or more, and hospitals and static clinics run by NGOs. From these frames, a total of 1,596 facilities were sampled in 2014 and 1,600 in 2017, respectively. Among these, 1,548 facilities from the 2014 BHFS and 1,524 facilities from the 2017 BHFS were interviewed, which are included in the sample of availability. For the analysis of readiness and statistical modelling, 1,260 facilities from the 2014 BHFS and 1,359 facilities from the 2017 BHFS that provided modern family planning services were used to identify associated factors with readiness scores. **Fig 1** illustrates the sampling process and selection of facilities included in the surveys.

### Measures

#### Outcome variables.

The outcome variables were the availability of health facilities offering modern family planning services and the readiness of facilities to provide these services. Service availability is defined as whether a facility offer any modern family planning method [contraceptive pills (combined or progestin-only), injectables (progestin only), one-rod or two-rod implants, intrauterine contraceptive devices (IUCDs), male condoms, female sterilization (tubal ligation) or male sterilization (vasectomy), and emergency contraceptives], prescribing it for clients to obtain elsewhere, or counselling clients about it [7]. The facility readiness was assessed by the Service Availability & Readiness Assessment (SARA) manual [29]. The SARA manual is a globally recognized, reliable tool for assessing health facilities’ capability to provide services, including family planning, child health services, obstetric care, non-communicable diseases, and others [29]. The modern family planning readiness of health facilities was evaluated using country-specific indicators under three domains: (a) staff and guidelines; (b) equipment and supplies; and (c) medicines and commodities. The selection of the indicators was determined based on their inclusion in the BHFS and adherence to the SARA manual [29]. A composite readiness score was calculated by summing all tracer indicators across three domains using a weighted additive approach, which assigned equal weights (33.3%) to each domain while adjusting for the variation in the number of tracer indicators in each domain [30,31]. The total readiness score was standardized to a maximum of 100 [30,31]. [30] In this analysis, readiness was calculated for different types of modern family planning methods (SARCs, LARCs, and PMs) and then aggregated into an overall readiness score, following SARA guidelines. The details of the SARA tracer indicators used in this study are presented in S1 Table. In the readiness calculation of IUCDs, Community Clinics were excluded, since they do not have the provision to offer such services, while for implants and PMs (tubal ligation and vasectomy), Union Health and Family Welfare Centers, Union Subcenters/Rural Dispensaries, and Community Clinics were excluded [7]. This adjustment in the readiness calculation ensures that readiness scores accurately reflect the readiness of facilities actually providing modern family planning services, consistent with previous studies [32].

#### Explanatory variables.

The explanatory variables selected in this analysis were based on previous literature [17,20], including facility type [District Hospital (DH), Maternal and Child Welfare Center (MCWC), Upazila Health Complex (UHC), Union Health and Family Welfare Center (UHFWC), Union Subcenter/Rural Dispensary (USC/RD), Community Clinic (CC), NGO Clinic/Hospital and Private Hospital], routine quality assurance (QA) activities (performed, not performed); external supervision (received, not received); user fees (yes, no); 24-hours staff coverage (available, not available); system of reviewing client feedback (available, not available); family planning service provision (regular, not regular); location of facility (urban, rural); division (Dhaka, Barishal, Chattogram, Khulna, Rajshahi, Rangpur, Sylhet and Mymensingh). Details of the operational definition of the explanatory variables are given in the S2 Table.

### Statistical analysis

Data analysis was performed using Stata version 17.0 (StataCorp, College Station, Texas, USA). We employed sample weights, guaranteeing the survey’s representativeness. The Stata “Svyset” command was utilized to handle the complex survey design. Descriptive statistics were used to show health facility characteristics, including the availability of modern family planning services. Weighting was applied for all estimates to adjust for disproportionate sampling and non-response. Notably, we analyzed each data set separately rather than combining them. We calculated the variance inflation factor (VIF) to examine multicollinearity among the explanatory variables. A proportion test was conducted to assess a significant change between 2014 and 2017. Incidence Rate Ratios (IRRs) were derived from the Poisson regression model to quantify the association between explanatory variables and modern family planning service readiness, as the outcome variable, representing the number of tracer indicators available in the facilities, was a count variable. IRR was presented with a 95% confidence interval (CI), and a P-value of less than 0.05 was defined as a level of significance. Additionally, ArcGIS version 10.8 was used to prepare the map of the availability and readiness of modern family planning services across different divisions of Bangladesh. A divisional shapefile was used to prepare the map layout, which was obtained from the Humanitarian Data Exchange (HDX), a platform managed by the United Nations Office for the Coordination of Humanitarian Affairs [33].

### Ethics statement and consent to participate

The datasets analyzed in this study were accessed with permission from the Demographic and Health Surveys Program following standard protocol. The protocols and questionnaires of the Bangladesh Health Facility surveys have been reviewed and approved by the ICF Institutional Review Board (IRB) and the National Research Ethics Committee of the Bangladesh Medical Research Council (BMRC). Since we performed a secondary analysis, our study did not require additional ethical approval. In terms of participant consent, all adult participants (aged 18 years or above) gave their written informed consent during the survey. For participants under the age of 18, parental or guardian consent was obtained in addition to the minor’s consent.

## Results

### Distribution of health facilities

A total of 1,548 facilities from the 2014 BHFS and 1,524 from the 2017 BHFS were included. CCs accounted for the majority of facilities (65.3% in 2014 and 66.4% in 2017), followed by UHFWCs (17.2% in 2014 and 16.4% in 2017) and USC/RDs (7% in 2014 and 7.3% in 2017). Meanwhile, DHs comprised the lowest percentage of total facilities (0.3% in both years). Very few of the facilities performed routine QA activities (17.5% in 2014 and 36.5% in 2017) and ensured 24-hour staff coverage (12% in 2014 and 15.7% in 2017). Most facilities were located in rural areas (91.6% in 2014 and 92.9% in 2017), with the highest proportion in the Dhaka division (27.2% in 2014 and 19.9% in 2017) (**Table 1**).

**Table 1:** Percentage distribution (weighted) of surveyed facilities in 2014 and 2017.

Variable	n (%)	
	2014	2017
**Facility type**		
District Hospital (DH)	5 (0.3)	5 (0.3)
Upazila Health Complex (UHC)	35 (2.2)	32 (2.1)
Maternal and Child Welfare Center (MCWC)	8 (0.5)	7 (0.5)
Union Health and Family Welfare Center (UHFWC)	266 (17.2)	250 (16.4)
Union Subcenter/Rural Dispensary (USC/RD)	108 (7)	111 (7.3)
Community Clinic (CC)	1010 (65.3)	1012 (66.4)
NGO Clinic/Hospital	81 (5.2)	64 (4.2)
Private Hospital	36 (2.3)	43 (2.8)
**Routine quality assurance activities**		
Not performed	1264 (82.5)	959 (63.5)
Performed	268 (17.5)	550 (36.5)
**External supervision**		
Not received	53 (3.4)	19 (1.2)
Received	1495 (96.6)	1505 (98.8)
**User fees**		
No	1387 (89.6)	860 (56.4)
Yes	161 (10.4)	664 (43.6)
**24-hour staff coverage**		
Not available	1362 (88)	1285 (84.3)
Available	186 (12)	239 (15.7)
**System for reviewing Client feedback**		
Not available	1116 (72.1)	911 (59.8)
Available	432 (27.9)	613 (40.2)
**Family planning service provision**		
Not regular	323 (25.2)	305 (22.2)
Regular	958 (74.8)	1072 (77.8)
**Location of facility**		
Urban	130 (8.4)	108 (7.1)
Rural	1418 (91.6)	1416 (92.9)
**Division**		
Dhaka	421 (27.2)	304 (19.9)
Barishal	116 (7.5)	113 (7.4)
Chattogram	287 (18.6)	288 (18.9)
Khulna	197 (12.7)	187 (12.3)
Rajshahi	224 (14.5)	220 (14.4)
Rangpur	205 (13.3)	193 (12.7)
Sylhet	97 (6.2)	96 (6.3)
Mymensingh	–	123 (8)

### Modern family planning service availability

**Fig 2:**
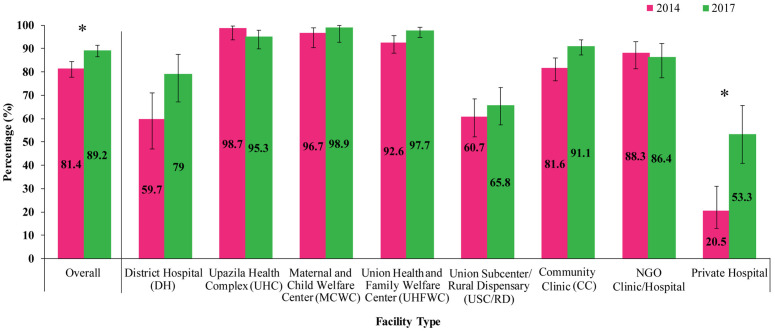
Facilities offering modern family planning services by survey years and facility types, 2014 and 2017. Asterisks (*) represent statistical significance (p-value < 0.05).

Between 2014 and 2017, the availability of facilities offering modern family planning services increased from 81.4% (95% CI: 77.8, 84.5) to 89.2% (95% CI: 87.5, 90.6). Between 2014 and 2017, a significant increase in modern family planning service availability was observed in private hospitals (20.5% to 53.3%). Conversely, UHCs and NGO clinics/hospitals experienced slight declines, from 98.7% to 95.3% and 88.3% to 86.4%, respectively. Other facility types, including DHs, MCWCs, UHFWCs, and CCs, showed slight increases in the availability of modern family planning services (**Fig 2**).

**Fig 3:**
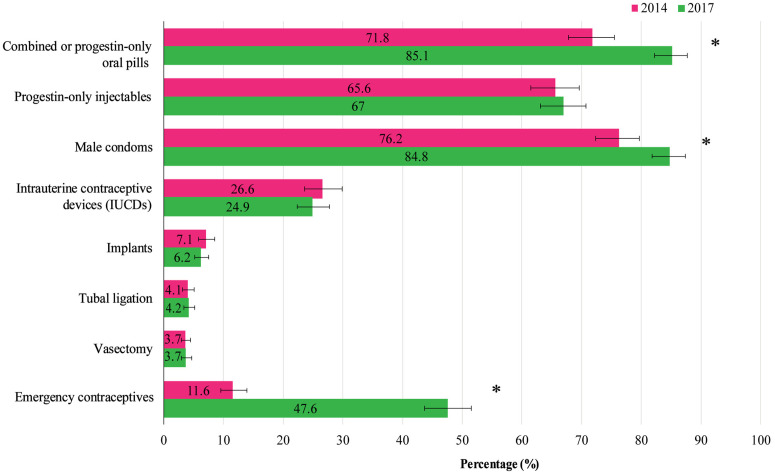
Availability of modern family planning methods in 2014 and 2017. Asterisks (*) represent statistical significance (p-value < 0.05).

The changes in the availability of modern family planning methods between 2014 and 2017 are shown in **Fig 3**. There was a significant increase in combined or progestin-only oral pills (71.8% to 85.1%) and male condoms (76.2% to 84.8%). Progestin-only injectables showed a slight increase from 65.6% to 67%. Most notably, the availability of emergency contraceptives increased significantly, rising from 11.6% in 2014 to 47.6% in 2017. In contrast, the availability of LARCs decreased slightly (IUCDs: 26.6% to 24.9%; implants: 7.1% to 6.2%). The availability of PMs (tubal ligation and vasectomy) remained relatively unchanged during this period.

**Fig 4:**
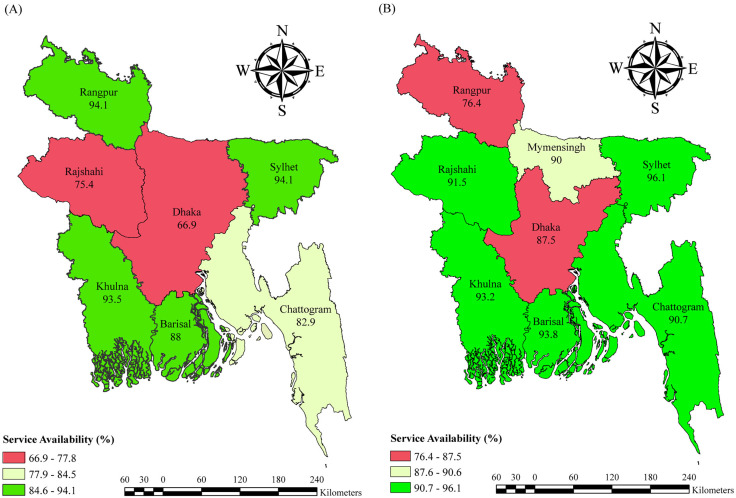
Facilities offering modern family planning services by division in 2014 (A) and 2017 (B).

The temporal variations in facilities with modern family planning service availability across divisions between 2014 and 2017 are depicted in **Fig 4**. Facilities from the Dhaka and Rajshahi divisions demonstrated a sharp increase from 66.9% to 87.5% and from 75.4% to 91.5% respectively. However, the availability of modern family planning services dropped in facilities in the Rangpur (94.1% to 76.4%) and Khulna (93.5% to 93.2%) divisions.

### Modern family planning service readiness

**Fig 5:**
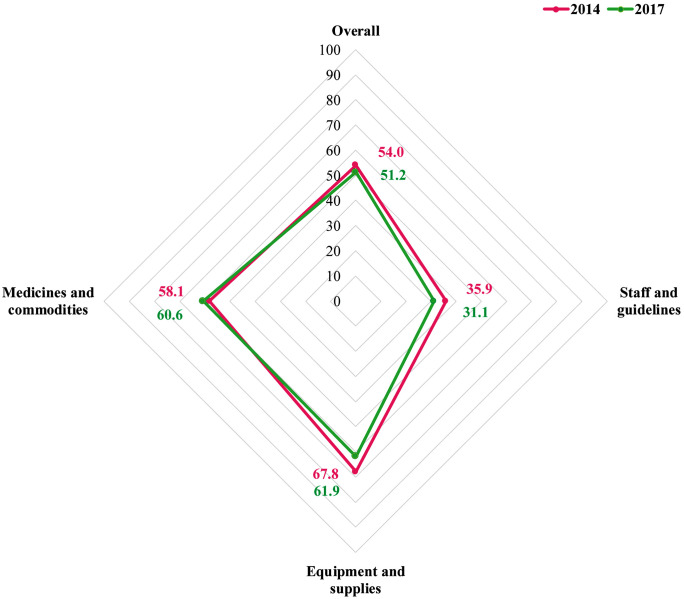
Overall and domain-specific mean readiness score by survey years, 2014 and 2017.

**Fig 5** presents the mean readiness score of the health facility for providing modern family planning services in 2014 and 2017. The overall mean readiness score declined slightly, from 54 (95% CI: 52.3, 55.7) in 2014 to 51.2 (95% CI: 49.7, 52.7) in 2017. The mean health facility readiness score across different domains showed notable variations between 2014 and 2017. Facility readiness score in staff and guidelines declined from 35.9 (95% CI: 33.1, 38.8) in 2014 to 31.1 (95% CI: 28.4, 33.8) in 2017. A significant decrease was observed in the facility readiness score for equipment and supplies [67.8 (95% CI: 65.4, 70.2) to 61.9 (95% CI: 60, 63.7)] over the same period. In contrast, facility readiness in medicines and commodities showed a slight increase, rising from 58.1 (95% CI: 56, 60.2) in 2014 to 60.6 (95% CI: 58.6, 62.7) in 2017. The distribution of overall and domain-specific mean readiness scores of facilities by background characteristics in 2014 and 2017 is provided in S3 Table.

**Fig 6:**
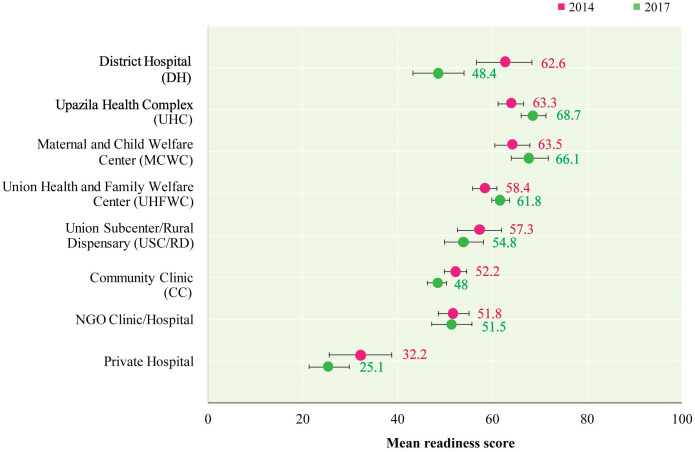
Mean readiness score of modern family planning service by facility type in 2014 and 2017.

In terms of facility type, DHs showed a significant decline in overall readiness score from 62.6 (95% CI: 56.7, 68.4) in 2014 to 48.4 (95% CI: 43, 53.9) in 2017. There was an increase in the readiness score of modern family planning services in UHCs (63.3 to 68.7), MCWCs (63.5 to 66.1), and UHFWCs (58.4 to 61.8) between 2014 and 2017. Meanwhile, CCs, NGO clinics/hospitals, and private hospitals showed a decline in readiness score over the same period (Fig 6 and S3 Table).

**Fig 7:**
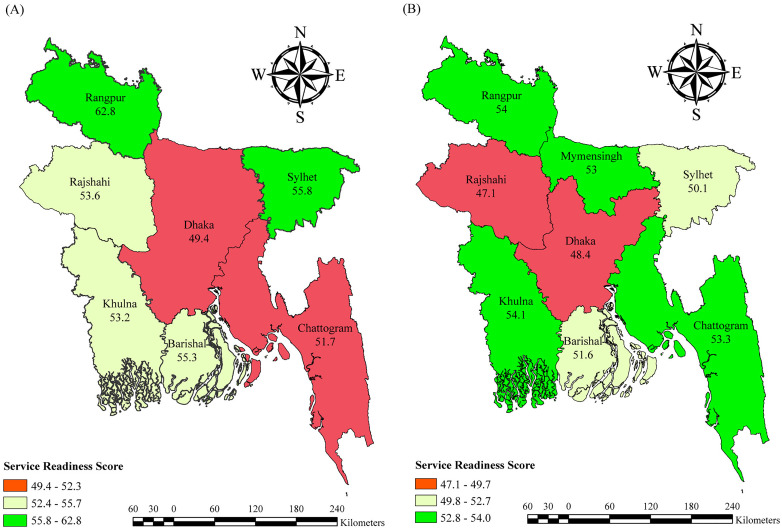
Modern family planning service readiness score by division in 2014 (A) and 2017 (B).

The mean health facility readiness score did not change significantly across divisions except in Rangpur, where it declined from 62.8 to 54 between 2014 and 2017. However, slight increases were observed in facilities in Chattogram (51.7 to 53.3) and Khulna (53.2 to 54.1). Overall, facilities in the Dhaka division scored lower than the mean readiness in both 2014 and 2017 (**Fig 7**).

### Factors associated with modern family planning service readiness

The factors associated with the readiness of health facilities offering modern family planning services are presented in **Table 2**. Considering facility types, UHCs showed 24% (IRR: 1.24; 95% CI: 1.07, 1.43) and 71% (IRR: 1.71; 95% CI: 1.48, 1.99) higher readiness score compared to district hospitals in 2014 and 2017, respectively. MCWCs showed no significant difference in readiness in 2014, but by 2017 demonstrated 63% (IRR: 1.63; 95% CI: 1.38, 1.93) higher readiness. UHFWCs and USC/RDs consistently showed low readiness scores compared to DHs over the years. CCs also demonstrated 88% (IRR: 0.12; 95% CI: 0.1, 0.14) and 85% (IRR: 0.15; 95% CI: 0.13, 0.18) lower readiness compared to DHs in 2014 and 2017, respectively. Private hospitals also had lower readiness compared to DHs in both survey years.

**Table 2:** Factors associated with facility readiness for providing modern family planning services in 2014 and 2017.

Variable	IRR (95% CI)	
	2014	2017
**Facility type**		
District Hospital (DH)	Reference	Reference
Upazila Health Complex (UHC)	1.24 (1.07, 1.43)**	1.71 (1.48, 1.99)***
Maternal and Child Welfare Center (MCWC)	1.14 (0.96, 1.34)	1.63 (1.38, 1.93)***
Union Health and Family Welfare Center (UHFWC)	0.36 (0.31, 0.43)***	0.52 (0.45, 0.61)***
Union Subcenter/Rural Dispensary (USC/RD)	0.33 (0.28, 0.39)***	0.45 (0.38, 0.54)***
Community Clinic (CC)	0.12 (0.1, 0.14)***	0.15 (0.13, 0.18)***
NGO Clinic/Hospital	0.72 (0.63, 0.84)***	0.87 (0.75, 1.02)
Private Hospital	0.51 (0.42, 0.62)***	0.57 (0.49, 0.67)***
**Routine quality assurance activities**		
Not performed	Reference	Reference
Performed	1.06 (1.02, 1.1)**	1.04 (1, 1.08)*
**External supervision**		
Not received	Reference	Reference
Received	0.92 (0.84, 1.01)	0.65 (0.57, 0.74)***
**User fees**		
No	Reference	
Yes	0.96 (0.91, 1.02)	1.01 (0.97, 1.05)
**24-hour staff coverage**		
Not available	Reference	Reference
Available	1.07 (1.02, 1.12)**	1.05 (1.01, 1.1)*
**System for reviewing client feedback**		
Not available	Reference	Reference
Available	1.12 (1.08, 1.16)***	1.08 (1.04, 1.12)***
**Family planning service provision**		
Not regular	Reference	Reference
Regular	1.19 (1.14, 1.24)***	1.06 (1.02, 1.11)**
**Location of facility**		
Urban	Reference	Reference
Rural	0.9 (0.85, 0.95)***	0.81 (0.77, 0.86)***
**Division**		
Dhaka	Reference	Reference
Barishal	1.08 (1.01, 1.14)*	0.99 (0.93, 1.06)
Chattogram	0.93 (0.89, 0.98)**	1.02 (0.97, 1.06)
Khulna	1.01 (0.96, 1.07)	1.05 (1, 1.11)
Rajshahi	1 (0.95, 1.06)	1.01 (0.96, 1.06)
Rangpur	1.09 (1.04, 1.15)**	1.06 (1.00, 1.12)*
Sylhet	1.02 (0.96, 1.09)	0.95 (0.89, 1.02)
Mymensingh	–	1.03 (0.97, 1.1)

* p-value < 0.05

** p-value < 0.01

*** p-value < 0.001

Facilities with routine quality assurance activities [IRR: 1.06 (95% CI: 1.02, 1.1) in 2014 and IRR: 1.04 (95% CI: 1, 1.08) in 2017], 24-hour staff coverage [IRR: 1.07 (95% CI: 1.02, 1.12) in 2014 and IRR: 1.05 (95% CI: 1.01, 1.1) in 2017], and having a system for reviewing client feedback (IRR: 1.12 (95% CI: 1.08, 1.16) in 2014 and [IRR: 1.08 (95% CI: 1.04, 1.12) in 2017] demonstrated higher readiness scores in both survey years. Facilities that regularly provided family planning services were also associated with higher readiness, though the effect declined from 19% (IRR: 1.19; 95% CI: 1.14, 1.24) in 2014 to 6% (IRR: 1.06; 95% CI: 1.02, 1.11) in 2017. In contrast, external supervision showed no significant association in 2014, but in 2017 was linked to a 35% (IRR: 0.65; 95% CI: 0.57, 0.74) lower readiness score.

Facilities located in rural areas had 10% (IRR: 0.9; 95% CI: 0.85, 0.95) lower readiness in 2014 and 19% (IRR: 0.81; 95% CI: 0.77, 0.86) lower readiness in 2017 compared to facilities in urban areas. Considering division-level variation, Barishal (IRR: 1.08; 95% CI: 1.01, 1.14) had significantly higher readiness in 2014 compared to Dhaka, while Chattogram showed 7% (IRR: 0.93; 95% CI: 0.89, 0.98) lower readiness. Rangpur had 9% (IRR: 1.09; 95% CI: 1.04, 1.15) and 6% (IRR: 1.06, 95% CI: 1, 1.12) higher readiness compared to Dhaka in 2014 and 2017, respectively.

## Discussion

Modern family planning is essential to safeguard the health of women and their babies, as well as improve the economic stability of families [34]. Analyzing BHFS data from 2014 and 2017, we found that while the availability of modern family planning services significantly increased across facilities, there was a slight decline in the overall facility readiness. Modern family planning service readiness was significantly influenced by the type of facility, the facility’s regular QA activities, 24-hour staff coverage, system for reviewing client feedback, the frequency of family planning service provision, and the area of the facility. We also identified regional disparities in facility readiness for modern family planning services. To the best of our understanding, this is the first study to evaluate changes in the availability and readiness of modern family planning services at the national and sub-national levels, as well as across various health facility types in Bangladesh, using data from the nationally representative BHFS 2014 and 2017.

We found the majority of the health facilities in Bangladesh offering modern family planning services with a slight increase between 2014 and 2017. An increase in the availability of modern family planning services over time has also been reported in studies conducted in other countries, such as the Democratic Republic of Congo, India, and the United States [35–37]. The increase in the availability of the modern family planning service in Bangladesh may be due to the government’s commitment to improving reproductive health services as part of its national health strategy. The Directorate General of Family Planning (DGFP) under the MOHFW is the main government body delivering family planning services in Bangladesh through a wide network of UHFWCs, where Family Welfare Visitors (FWVs) provide counselling and SARCs, including IUCDs [38]. Furthermore, the relatively high availability of modern family planning services may be attributed to the success of Bangladesh’s vertical family planning program and its dedicated service delivery mechanism [39]. This commitment often includes increased awareness campaigns, investments in healthcare infrastructure, the training of healthcare providers, and the integration of family planning services into broader MCH programs [40].

We also found that the availability of SARCs, such as combined and progestin-only oral contraceptive pills and male condoms, was high, which corresponds to a previous study conducted in Bangladesh [41]. In Bangladesh, oral contraceptive pills and male condoms are the most popular modern family planning methods [14]. Research across multiple LMICs also found high availability of oral contraceptives and male condoms in health facilities [42,43], where health systems tend to prioritize SARCs due to lower cost, simpler logistics, and less requirement for trained providers [44,45]. In contrast, LARCs such as IUCDs and implants had low availability in health facilities in Bangladesh. This finding was consistent with previous studies [32,46]. The DGFP carries out various behavioral change communication activities to increase the use of LARCs and PMs. However, there is a lack of coordination between family planning workers and health facilities. In Bangladesh, family planning workers work under the DGFP, and health facilities function under the DGHS [20]. Family planning workers at the community level raise awareness about LARCs and PMs among rural women and counsel eligible couples on family planning, but they only provide referrals for LARCs rather than delivering the methods themselves [47].

Despite improvements in the availability of modern family planning services, overall facility readiness was low and showed a decline between 2014 and 2017. A previous study on public facilities in Bangladesh also reported low preparedness to provide general family planning services [46]. Evidence from a study conducted in ten countries in South Asia and Sub-Saharan Africa shows that the majority of facilities failed to meet at least 75% of the standards for family planning service readiness [17]. In contrast to our findings, a study conducted in Nepal reported that facility readiness improved in parallel with family planning service availability [20]. In Bangladesh, challenges such as limited funding for healthcare and insufficient training opportunities for staff could contribute to the observed decline in facility readiness despite improvements in availability [48]. Additionally, unfair distribution of logistics, a shortage of healthcare providers, inadequate supervision and training, and service provider absenteeism could all contribute to this low readiness [18,48]. In Bangladesh, efforts to address the shortage of trained staff and guidelines, as well as equipment and supplies, may be warranted to ensure equitable access to a range of modern family planning services [49].

Our study found that there is a decline in the overall readiness score of modern family planning services in DHs. However, UHCs and MCWCs had significantly higher readiness scores compared to the DHs. Improvements in these facilities are not surprising, as all DHs, MCWCs, and UHCs are expected to provide all types of modern family planning services as per the Bangladesh Essential Health Service Package (ESP), aiming to reduce maternal mortality in Bangladesh [39]. As the first-level referral facility, UHCs should be ready to provide 24/7 family planning services. Furthermore, UHCs are also considered a strategic location by the government and have started working to strengthen the modern family planning services with the necessary human resources, infrastructure, and equipment [39,50]. However, USC/RDs and CCs had lower readiness to offer modern family planning services. The low readiness for modern family planning services provision of these facilities is due to the fact that they do not provide LARCs or PMs [32]. Their services are usually limited to SARCs (like pills and condoms), which reduces their readiness in modern family planning service provision [32]. Union-level public facilities may not be prepared due to an inefficient budget allocation policy, which could result in shortages and unequal distribution of medical supplies [7,26]. Additionally, we found that public facilities had higher readiness compared to private/NGO-managed facilities, which corresponds to earlier research done in low-resource settings, which found private/NGO-managed facilities had a lower readiness score than publicly managed facilities [17,51]. Another study from Bangladesh found that public facilities generally demonstrate higher readiness for LARCs and PMs compared to private facilities [18]. In Bangladesh, government investment and resource allocation heavily favor the public sector and provide commodities, training, and infrastructure support [23]. On the other hand, regulatory hurdles, a lack of government assistance, and an excessive emphasis on business-driven services may prevent private institutions from providing modern family planning services, particularly long-acting reversible contraceptives [52,53].

Facilities that perform routine QA activities had significantly high readiness scores to provide modern family planning services, which was consistent with a study conducted in 10 LMICs [17]. In Nepal, facilities performing routine QA procedures had higher readiness scores to provide family planning and MCH services [54]. QA is a system that keeps track of the quality of services delivered by identifying issues and implementing corrective measures to resolve them [55]. QA also entails examining and auditing all necessary elements, including medicines and diagnostic capabilities, human resources and guidelines, and necessary commodities to ensure delivery of integrated, high-quality family planning services [17]. Similarly, facilities having a system for gathering and evaluating clients’ feedback also showed greater readiness for modern family planning services. A previous study in Nepal found that facilities that review clients’ opinions had better readiness to provide family planning and MCH services [54]. This is plausible as the facilities that had the system in place for collecting and evaluating client opinions were able to get feedback from clients and perform troubleshooting exercises. Another study found that health facilities with a system for collecting and reviewing client feedback were better prepared to provide general health care than those without such a system [51]. We found that facilities that provide family planning services regularly demonstrate significantly higher readiness compared to those offering services less frequently. A previous study also found that the median number of days family planning services are offered per month is significantly associated with a higher readiness score [17]. This consistent availability likely contributes to better stock management, continuous presence of trained healthcare providers, and strict adherence to service guidelines, all of which enhance facility preparedness [20,56]. Daily service provision also facilitates improved provider-client interactions, fostering higher quality care and responsiveness to client needs [57].

A significant urban-rural disparity was evident in modern family planning service readiness, which was consistent with findings from previous studies in Bangladesh that reported lower readiness of health facilities to provide LARCs in rural areas compared to urban settings [18]. Family planning readiness was lower in rural health facilities compared to urban ones, mainly due to resource limitations and service delivery challenges [58]. Additionally, geographic barriers and a weaker health system support in rural areas reduced access to and continuity of family planning services [24,59]. Our study found regional variation in the modern family planning readiness in terms of division. Facilities from the Rangpur divisions had significantly higher readiness to provide modern family planning services compared to the Dhaka division in 2014 and 2017. A previous study in Bangladesh also found that Rangpur had higher readiness to provide LARCs [18]. Furthermore, Rangpur has a relatively higher acceptance rate of modern contraceptive use [60], which likely drives greater demand and, in turn, supports higher facility readiness in the region [24]. In contrast, a previous study in Nepal found that regional variation in the area of the facility did not affect the family planning readiness score [20]. This geographical variation echoes findings that underscore disparities in healthcare infrastructure and resource allocation across regions [18,20]. Factors contributing to these differences may include varying levels of government investment, disparities in healthcare workforce distribution, and differential access to training and technical assistance [19,55].

### Strengths and limitations

The data analyzed in this study, which were nationally representative, were collected using standardized questionnaires. Therefore, the findings can be extrapolated to the broader health system in Bangladesh. Furthermore, identifying factors associated with facility readiness to provide modern family planning services and changes in the level of readiness across different variables, such as facility types and divisions, provides policymakers and stakeholders with targeted information to address gaps and improve service provision. Moreover, due to the complex sampling process used to acquire SPA data, sample weights and cluster effects were taken into consideration in the current analysis, so the estimates are comparable to those of other SPA surveys. The readiness assessment accounted for the specific equipment and supplies required for all types of modern family planning methods, including SARCs, LARCs, and PMs, thereby providing comprehensive readiness estimates. Finally, we performed a comparative analysis using the BHFS from 2014 and 2017 to capture how modern family planning service readiness has evolved throughout Bangladesh’s healthcare facilities.

This study has several limitations worth considering. Firstly, the availability of modern family planning services and readiness indicators was measured based on the presence on the day of the visit, which provides a cross-sectional snapshot. Secondly, the data on service availability is based on the facility’s self-reported status, while data collectors physically observed the facility to confirm that all essential components were available and functioning as intended. Consequently, using self-reported data may introduce social desirability and recall biases, which could interfere with the accuracy of the responses. Thirdly, the BHFS 2014 and BHFS 2017 datasets that were utilized in the current study are comparatively outdated. Since the most recent dataset was unavailable, the findings may not fully reflect the current situation. Finally, we must recognize that differences in the availability and readiness of modern family planning services may reflect challenges related to the health system, such as health governance and funding. Due to insufficient data, we were unable to delve deeper into and pinpoint the underlying issues, which will require further investigation in subsequent research.

## Conclusion

Our study demonstrates an increase in the availability of modern family planning services in health facilities in Bangladesh. In terms of facility readiness, a decline was observed over time, reflecting shortages of trained staff and limited availability of family planning equipment for the provision of LARCs and PMs. Facility readiness for modern family planning services differed significantly with respect to the supervision and service delivery domains. There was an urban-rural and divisional disparity in the modern family planning service readiness. It is essential to invest in training healthcare providers and ensure a steady supply of essential family planning equipment and supplies. Additionally, implementing routine quality assurance activities and increasing investments in rural facilities can enhance service readiness. Fostering public-private partnerships can bolster the availability and readiness of modern family planning services, ensuring that both sectors are effectively utilized to meet the SDG goals and contribute to global reproductive health targets.

## Supporting information

S1 Table Tracer indicators for modern family planning services readiness. (DOCX)

S2 Table Operational definition of the explanatory variables used in the study. (DOCX)

S3 Table Overall and domain-specific mean readiness score of modern family planning services by background characteristics in 2014 and 2017. (DOCX)

S1 File Datasets used in the study. (XLSX)
