# Supplementary material for: Assessing changes in the availability and readiness of health facilities to provide modern family planning services in Bangladesh: Insights from Bangladesh Health Facility Surveys, 2014 and 2017
Source: PLoS One. 2025 Nov 14;20(11):e0334520. doi: 10.1371/journal.pone.0334520 (PMC12617875; doi:10.1371/journal.pone.0334520)
Supplement: S1 Table — (DOCX) [file pone.0334520.s001.docx]

**S1 Table. Tracer indicators for modern family planning service readiness.**

| **Domain** | **Tracer indicators** | | | | | |
| --- | --- | --- | --- | --- | --- | --- |
|  | **Short-acting reversible contraceptives**  **(SARCs)** | **Long-acting reversible contraceptives (LARCs)** | | **Permanent methods**  **(PMs)** | |  |
|  |  | IUCDs | Implants | Tubal Ligation | Vesectomy |  |
| **Staff and guidelines** | - Guidelines on family planning ^a^ - Staff trained in basic modern family planning services in the last 24 months ^b^ | - Guidelines on family planning ^a^ - Staff trained in IUCDs in the last 24 months | - Guidelines on family planning ^a^ - Staff trained in implants in the last 24 months | - Guidelines on family planning ^a^ - Staff trained in performing tubal ligation in the last 24 months | - Guidelines on family planning ^a^ - Staff trained in performing vasectomy in the last 24 months |  |
| **Equipment and supplies** | - Blood pressure (BP) apparatus ^c^ - Stethoscope - Model for showing condom use - FP-specific visual aid^d^ | - Examination light (flashlight/torch) - Examination bed/couch - Pelvic model for IUCDs - Sterile gloves - Antiseptic solution (e.g., povidon iodine) - Sponge holding forceps - Gauze pad/cotton wool - Gallipot - Cusco Vaginal Speculum (S + M + L) - Tenaculm (volsellum forceps) - Uterine sound - Straight artery forceps - Straight cutting scissors - Bucket (for waste disposal) - IUCD in sterile packaging - Autoclave/IUCD sterilizer/sterilizer | - Examination light (flashlight/torch) - Examination bed/couch - Sterile gloves - Antiseptic solution (e.g., povidon iodine) - Sponge holding forceps - Gauze pad/cotton wool - Gallipot - Sterile syringe and needle - Canula and torcher for inserting implant - Implant pack - Scalpel with blade - Arm rest/side table - Marker pen - Band aid - Normal bandage/ butterfly bandage - Elastomeric mattress dressing - Surgical drapes - Handwashing soap | - Operating theatre (OT) table - OT light - Autoclave/sterilizer - BP handle - Needle holder - Babock tissue forceps - Long straight artery forceps - Curved mosquito artery forceps - Alice tissue forceps - Plain dissecting forceps - Mayo scissors - Sponge holding forceps - Retractor - Sterile chromic CATGUT/PJA - Lifter jar with lifter - Tubectomy kit - Instrument trolley - Cutting curved needle - Provider gown - Trolley sheet - Draw sheet - Cap - Mask - Gauze/cotton - Antiseptic solution (e.g., povidon iodine) - Surgical gloves - Sterile syringe - Surgical blade | - OT table - OT light - Autoclave/sterilizer - Non-surgical vasectomy kit - Ring forceps - Vas dissecting forceps - Small surgical scissors - Condom - Galipot - Lifter jar with lifter - Provider gown - Trolley sheet - Instrument trolley - Cap - Mask - Gauze/cotton - Sterile gloves - Sterile syringe - Silk thread - Antiseptic solution (e.g., povidon iodine) |  |
| **Medicines and commodities** | - Combined oral contraceptive pills - Progestin-only oral pills - Progestin-only injectables - Male condoms - Emergency contraceptive pills | - Paracetamol or Ibuprofen - Iron folic | - Local anesthetic (e.g., 1% Lidokaine) - Paracetamol | - Inj. Atropine sulphate (0.6 mg/ml) - Inj. Promethazine (1.2 mg/ml) - Inj. Pethidine (2.5 mg/ml) - Inj. Xylocaine (1%) - Diazepam tablets (5 mg) - Antibiotic (Ciprofloxacin or Azithromycin) - Paracetamol tablets - Iron + Folic acid tablets | - Inj. Xylocaine (1%) - Antibiotic (Ciprofloxacin or Azithromycin) - Paracetamol tablets - Vitamin B-Complex tablets |  |

^a^ National guidelines/manual or any other guidelines/instructions/job aid/checklist on family planning.

^b^ At least one staff trained in general counselling and either clinical management of family planning methods, post-partum family planning, injectable contraceptives, emergency contraceptive pill, or other training on family planning services in the last 24 months.

^c^ A functioning digital blood pressure apparatus or a manual sphygmomanometer with a stethoscope.

^d^ Flip charts or leaflets
