# Supplementary material for: Assessing changes in the availability and readiness of health facilities to provide modern family planning services in Bangladesh: Insights from Bangladesh Health Facility Surveys, 2014 and 2017
Source: PLoS One. 2025 Nov 14;20(11):e0334520. doi: 10.1371/journal.pone.0334520 (PMC12617875; doi:10.1371/journal.pone.0334520)
Supplement: S2 Table — (DOCX) [file pone.0334520.s002.docx]

**S2 Table. Operational definitions of the explanatory variables used in the study.**

| **Explanatory**  **variable** | **Operational definition** |
| --- | --- |
| **Facility type** | Healthcare facilities are classified into District Hospital (DH), Maternal and Child Welfare Center (MCWC), Upazila Health Complex (UHC), Union Health and Family Welfare Center (UHFWC), Union Subcenter/ Rural Dispensary (USC/RD), Community Clinic (CC), NGO Clinic/Hospitals, and Private hospital, based on management and service provision. |
| **Routine quality assurance (QA) activities** | Facilities that conducted quality assurance (QA) activities, such as mortality reviews or register audits within the past 12 months, are categorized as performing QA activities. Those that did not are classified otherwise. |
| **External supervision** | External supervision is considered present if the facility received oversight from an upper-level facility or higher authority within the last six months. Facilities without such supervision are categorized as not received. |
| **User fees** | Facilities that require patients to pay for services are classified as 'yes,' while those providing services free of charge are classified as 'no.' |
| **24-hour staff coverage** | Continuous staff availability is determined by the presence of at least one health professional (medical specialist, medical officer, nurse, paramedic) 24/7, either on duty or on-call. If this coverage is missing, the facility is classified as lacking 24-hour coverage. |
| **System for reviewing client feedback** | A structured mechanism for gathering and reviewing patients' opinions about facility services qualifies as a client feedback system. Facilities without such a system fall into the "not available" category. |
| **Family planning service provision** | Provision of family planning services is classified as ‘regular’ if available on 20 or more days per month. If offered less frequently, they are categorized as ‘not regular.’ |
| **Location of facility** | Facilities are classified as urban or rural according to the definition provided in the Bangladesh Health Facility Survey (BHFS) report. |
| **Division** | Facilities are assigned to one of **eight divisions in Bangladesh: Dhaka, Barishal, Chattogram, Khulna, Rajshahi, Rangpur, Sylhet, and Mymensingh**, based on their geographic location. **However, in the BHFS 2014, Mymensingh was included under the Dhaka division, as it had not yet been established as a separate division.** |
