# Supplementary material for: Assessing changes in the availability and readiness of health facilities to provide modern family planning services in Bangladesh: Insights from Bangladesh Health Facility Surveys, 2014 and 2017
Source: PLoS One. 2025 Nov 14;20(11):e0334520. doi: 10.1371/journal.pone.0334520 (PMC12617875; doi:10.1371/journal.pone.0334520)
Supplement: S3 Table — (DOCX) [file pone.0334520.s003.docx]

**S3 Table. Overall and domain-specific mean readiness scores for modern family planning services by background characteristics in 2014 and 2017.**

| **Variable** | **Overall** | | **Staff and guidelines** | | **Equipment and supplies** | | **Medicines and commodities** | | |
| --- | --- | --- | --- | --- | --- | --- | --- | --- | --- |
|  | **Mean readiness score (95% CI)** | | **Mean readiness score (95% CI)** | | **Mean readiness score (95% CI)** | | **Mean readiness score (95% CI)** | | |
|  | **2014** | **2017** | **2014** | **2017** | **2014** | **2017** | | **2014** | **2017** |
| **Overall** | **54 (52.3, 55.7)** | **51.2 (49.7, 52.7)** | 35.9 (33.1, 38.8) | 31.1 (28.4, 33.8) | 67.8 (65.4, 70.2) | 61.9 (60, 63.7) | | 58.1 (56, 60.2) | 60.6 (58.6, 62.7) |
| **Facility type** | | | | | | | | | |
| District Hospital (DH) | 62.6 (56.7, 68.4) | 48.4 (43, 53.9) | 52.3 (43.9, 60.6) | 38.9 (31.1, 46.8) | 67.3 (59.5, 75.2) | 51.5 (45.2, 57.8) | | 68.2 (61.6, 74.7) | 54.9 (49.2, 60.5) |
| Upazila Health Complex (UHC) | 63.3 (60.7, 66) | 68.7 (66, 71.4) | 41.8 (37.2, 46.5) | 55.8 (51.7, 59.9) | 76.2 (72.6, 79.9) | 74.2 (70.9, 77.5) | | 71.9 (68.7, 75) | 75.9 (72.7, 79.1) |
| Maternal and Child Welfare Center (MCWC) | 63.5 (59.8, 67.2) | 66.1 (62.2, 70) | 50.5 (44.8, 56.2) | 51.9 (46, 57.8) | 71.5 (66.9, 76.2) | 73.1 (68.5, 77.8) | | 68.5 (64.6, 72.4) | 73.2 (68.8, 77.5) |
| Union Health and Family Welfare Center (UHFWC) | 58.4 (55.9, 61) | 61.8 (60, 63.7) | 36.3 (32.3, 40.3) | 44.8 (41.4, 48.1) | 74.8 (71.2, 78.5) | 69.2 (66.7, 71.7) | | 64.2 (61.5, 66.9) | 71.5 (69.3, 73.8) |
| Union Subcenter/Rural Dispensary (USC/RD) | 57.3 (52.6, 61.9) | 54.8 (50.7, 59) | 33.5 (26.9, 40.1) | 34.4 (27.8, 41.1) | 74 (68.1, 80) | 67.7 (63.4, 72) | | 64.2 (58.6, 69.8) | 62.4 (56.3, 68.5) |
| Community Clinic (CC) | 52.2 (49.8, 54.6) | 48 (46, 50.1) | 34.7 (30.5, 38.9) | 25.5 (21.8, 29.2) | 66.4 (62.9, 69.8) | 60.3 (57.8, 62.8) | | 55.6 (52.6, 58.7) | 58.3 (55.5, 61.1) |
| NGO Clinic/Hospital | 51.8 (48.5, 55) | 51.5 (48, 55) | 47.7 (42.7, 52.8) | 52.6 (47.1, 58.1) | 53.2 (48.9, 57.5) | 51.7 (47.9, 55.5) | | 54.3 (50.6, 58) | 50.2 (46.9, 53.5) |
| Private Hospital | 32.2 (25.6, 38.8) | 25.1 (20.8, 29.4) | 21.8 (10.4, 33.1) | 7.7 (2.2, 13.2) | 38.6 (30.3, 46.8) | 34.2 (28.1, 40.3) | | 36.1 (26.7, 45.6) | 33.4 (25.9, 40.9) |
| **Routine quality assurance activities** | | | | | | | | | |
| Not performed | 52.9 (51, 54.9) | 50.8 (48.9, 52.7) | 35 (31.7, 38.3) | 29.6 (26.4, 32.8) | 67 (64.3, 69.7) | 61.4 (59, 63.8) | | 56.8 (54.4, 59.1) | 61.3 (58.7, 63.8) |
| Performed | 59.3 (55.9, 62.7) | 52.1 (49.7, 54.5) | 41.9 (36.2, 47.6) | 33.6 (28.9, 38.3) | 72.1 (66.8, 77.3) | 63.2 (60.6, 65.8) | | 64 (58.7, 69.2) | 59.5 (56.1, 62.8) |
| **External supervision** | | | | | | | | | |
| Not received | 51.9 (44.5, 59.3) | 45.3 (38.9, 51.8) | 33.2 (20.5, 45.9) | 12.9 (-2.3, 28.1) | 69.3 (60.5, 78) | 56.8 (48.2, 65.5) | | 53.1 (40.9, 65.4) | 66.2 (53.9, 78.5) |
| Received | 54 (52.3, 55.8) | 51.3 (49.8, 52.8) | 36 (33.1, 39) | 31.3 (28.6, 34) | 67.8 (65.3, 70.3) | 61.9 (60.1, 63.8) | | 58.3 (56.1, 60.4) | 60.6 (58.5, 62.6) |
| **User fees** | | | | | | | | | |
| No | 54 (52.2, 55.9) | 52.5 (50.6, 54.5) | 34.8 (31.7, 37.9) | 32 (28.4, 35.7) | 68.7 (66.1, 71.4) | 63.6 (61.3, 65.9) | | 58.5 (56.3, 60.8) | 62 (59.3, 64.7) |
| Yes | 53.3 (50.6, 55.9) | 49.5 (47.2, 51.8) | 46.6 (40.4, 52.9) | 29.9 (26, 33.8) | 59.2 (55.4, 63) | 59.7 (56.7, 62.6) | | 54 (48, 60) | 58.9 (55.9, 61.9) |
| **24-hour staff coverage** | | | | | | | | | |
| Not available | 53.7 (51.8, 55.6) | 50.1 (48.3, 51.8) | 35.1 (31.9, 38.4) | 29.4 (26.3, 32.5) | 67.9 (65.2, 70.6) | 61.3 (59.2, 63.4) | | 58 (55.8, 60.3) | 59.4 (57.1, 61.8) |
| Available | 56.2 (53, 59.3) | 57.4 (55, 59.7) | 42.5 (38.8, 46.3) | 40.2 (36.1, 44.3) | 67.2 (63.1, 71.2) | 64.9 (62.3, 67.5) | | 58.8 (53.2, 64.4) | 67 (64.6, 69.5) |
| **System for reviewing client feedback** |  |  |  |  |  |  | |  |  |
| Not available | 52.5 (50.4, 54.5) | 49.9 (47.8, 52) | 32.9 (29.5, 36.4) | 28.3 (24.8, 31.8) | 66.8 (63.8, 69.7) | 60.6 (57.9, 63.3) | | 57.7 (55.3, 60) | 60.9 (58.1, 63.6) |
| Available | 57.9 (55, 60.8) | 53.2 (51.2, 55.2) | 43.8 (38.8, 48.8) | 35.4 (31.3, 39.4) | 70.6 (66.6, 74.7) | 63.8 (61.8, 65.8) | | 59.3 (54.7, 63.8) | 60.3 (57.4, 63.2) |
| **Family planning service provision** |  |  |  |  |  |  | |  |  |
| Not regular | 47.3 (43.7, 50.9) | 47.3 (43.6, 51) | 30.3 (24.9, 35.7) | 25.8 (20.1, 31.5) | 61.5 (56.1, 66.8) | 61 (57, 65) | | 50.1 (45.3, 54.8) | 55.2 (49.6, 60.7) |
| Regular | 56.1 (54.3, 58) | 52.3 (50.7, 53.9) | 37.8 (34.4, 41.2) | 32.6 (29.6, 35.6) | 69.9 (67.2, 72.6) | 62.1 (60, 64.2) | | 60.7 (58.5, 63) | 62.2 (60.1, 64.2) |
| **Location of facility** |  |  |  |  |  |  | |  |  |
| Urban | 54.4 (51.7, 57.2) | 50.4 (46.8, 54) | 43 (39.1, 46.9) | 40.9 (35.7, 46.2) | 60.2 (56.4, 63.9) | 55.4 (51.8, 59) | | 60.1 (57.3, 63) | 54.8 (51, 58.7) |
| Rural | 53.9 (52.1, 55.7) | 51.3 (49.7, 52.9) | 35.4 (32.3, 38.5) | 30.4 (27.6, 33.3) | 68.4 (65.8, 71) | 62.3 (60.4, 64.2) | | 58 (55.7, 60.2) | 61 (58.9, 63.2) |
| **Division** |  |  |  |  |  |  | |  |  |
| Dhaka | 49.4 (45.1, 53.8) | 48.4 (44.5, 52.3) | 36.7 (29, 44.3) | 29.1 (22.2, 36.1) | 58.2 (52, 64.4) | 58.5 (53.5, 63.5) | | 53.4 (47.5, 59.4) | 57.5 (52.8, 62.2) |
| Barishal | 51.7 (47.9, 55.6) | 53.3 (49.4, 57.3) | 36.4 (29.4, 43.4) | 35.8 (28.9, 42.8) | 62.4 (57.5, 67.2) | 61.7 (57, 66.3) | | 56.4 (51.9, 60.9) | 62.5 (58.1, 66.8) |
| Chattogram | 55.3 (52.1, 58.5) | 51.6 (47.1, 56) | 38.6 (32.3, 44.8) | 29.7 (22.6, 36.7) | 66.9 (62.4, 71.4) | 63.3 (57.5, 69) | | 60.4 (57.2, 63.7) | 61.7 (56.2, 67.2) |
| Khulna | 53.2 (49.3, 57.2) | 54.1 (50.5, 57.5) | 32.1 (25.6, 38.7) | 35.9 (29.4, 42.4) | 70.8 (65.3, 76.3) | 61.7 (57.7, 65.7) | | 56.8 (52, 61.6) | 64.3 (59.3, 69.3) |
| Rajshahi | 53.6 (48.6, 58.6) | 47.1 (43, 51.1) | 31.8 (23.7, 39.8) | 25.9 (18.9, 32.9) | 66.9 (59.8, 74) | 58.6 (53.5, 63.7) | | 62.2 (55.7, 68.7) | 56.6 (49.5, 63.7) |
| Rangpur | 62.8 (59.4, 66.1) | 54 (50.3, 57.7) | 38.2 (31.9, 44.6) | 31 (24.6, 37.4) | 85.8 (81.5, 90) | 69 (64.7, 73.3) | | 64.3 (60.3, 68.2) | 62 (55.9, 68.1) |
| Sylhet | 55.8 (51.7, 59.8) | 50.1 (47, 53.2) | 40.3 (32, 48.6) | 25.9 (19.5, 32.3) | 70.4 (64.1, 76.6) | 62.9 (58.4, 67.4) | | 56.6 (53.4, 59.8) | 61.5 (56.9, 66.1) |
| Mymensingh | - | 53 (48.4, 57.5) | - | 32.4 (22.7, 42) | - | 64.9 (60.2, 69.6) | | - | 61.8 (56, 67.5) |
